# Effects of Viscosity on Submerged Membrane Microfiltration Systems

**DOI:** 10.3390/membranes12080780

**Published:** 2022-08-14

**Authors:** Muna Pradhan, Md Abu Hasan Johir, Jaya Kandasamy, Harsha Ratnaweera, Saravanamuthu Vigneswaran

**Affiliations:** 1 Faculty of Engineering, University of Technology Sydney (UTS), P.O. Box 123, Ultimo, NSW 2127, Australia; 2 BMB Engineers, P.O. Box 7842, Baulkhan Hills, NSW 2153, Australia; 3 Faculty of Sciences & Technology (RealTek), Norwegian University of Life Sciences, P.O. Box 5003, NO-1432 Ås, Norway

**Keywords:** membrane filtration, viscosity, airflow, cake resistance, permeate flux, membrane fouling, transmembrane pressure (TMP)

## Abstract

Submerged microfiltration has a wide range of applications in water and wastewater treatment. Membrane fouling is a major problem, resulting in a severe decline in flux, high energy consumption and frequent membrane cleaning and replacement. The effect of viscosity was not previously studied under controlled conditions to relate it to the air scour. Hence, this study investigated the effect of viscosity on membrane fouling during the operation of submerged membrane microfiltration by adding predetermined amounts of glycerol to a kaolin clay suspension. The addition of glycerol increased the viscosity (from 0.001 to 0.003 Pa·s), resulting in a 3-fold higher transmembrane pressure (TMP) development. An increased airflow (air scour) rate by 3 fold (from 0.6 m^3^/m^2^/h to 1.8 m^3^/m^2^/h), reduced TMP development by 65%. Membrane fouling quickly developed during the initial stage of microfiltration operation. Therefore, special precautions to control fouling during the early stages of filtration could significantly enhance the operation of the microfilter. Higher airflow caused a reduction in average specific cake resistance, whereas higher viscosity increased this value.

## 1. Introduction

Membrane microfiltration is a pressure-driven separation process with many applications in water, wastewater treatment, chemical, and dairy industries. In practice, a significant problem associated with its application arises from membrane fouling, resulting in a severe decline in flux, high energy consumption, and frequent membrane cleaning and replacement [1]. Membrane fouling results from various physicochemical interactions between the suspension containing biomass and the membrane itself. Fouling studies are summarized in the literature [2,3,4,5]. The causes of membrane fouling can be classified into three categories: membrane and module characteristics, operating conditions and feed characteristics. Like conventional activated sludge processes, biomass viscosity in the membrane bioreactor is closely related to its mixed liquor suspended solids (MLSS) concentration and has been referred to as a foulant parameter. The viscosity of the suspension affects air bubble properties and other bubble-induced effects. In many industries, the feed viscosity can increase significantly during processing and particularly during increasing concentration caused by filtration. This directly affects membrane fouling. For example, in hot white pulp wastewater, the protein in the extracellular polymeric substance (EPS) is more than the polysaccharide causing the viscosity of the suspension to increase [6]. Changing the viscosity of the suspension can affect the treatment efficiency and stability of the reactor [7].

Excessive viscosity of the sludge mixture increases the likelihood of sludge adhering to the membrane surface, causing membrane fouling to accelerate. Hu [8] demonstrated that sludge with high viscosity is not easy to clean after being adsorbed to the membrane surface. This results in poor recovery of membrane flux.

Rosenberger et al. [9] observed that MLSS was a major parameter affecting the apparent viscosity of the suspension in membrane bioreactors. High MLSS concentrations can reduce the sludge loading rate, improve the treatment efficiency and increase the viscosity of the mixed liquor. Itonaga et al. [10] reported that the suspension viscosity tends to increase exponentially with the solids concentration. Hasar et al. [11] similarly found that the apparent viscosity of the sludge in a submerged membrane bioreactor increases exponentially with an increase in the solids content of the sludge. Le-Clech et al. [12] established an exponential relationship between MLSS concentration and viscosity for varying shear rates. Delrue et al. [13] observed an exponential relationship between mixed liquor viscosity and MLSS for different operating conditions. Similarly, Komesli and Gokcay [14] reported that the dynamic viscosity increased exponentially with an increasing MLSS concentration of the suspension in the membrane bioreactor for all temperatures.

The major concern of biomass viscosity in a membrane bioreactor is its detrimental effect on membrane fouling, air bubble properties and bubble-induced effects. To date, applying air bubbles into a submerged filtration system has proved effective, simple, and a low-cost technique to control fouling [15]. In an air-sparged microfiltration system, turbulence in the air bubble wake is responsible for accelerating mass transfer from the membrane surface back to the suspension. The strength and size of the air bubble wake depend on the bubble size. Larger bubbles produce a symmetric vortex in the wake region [16], so the secondary flow is much stronger for the larger bubbles. MLSS or viscosity modifies bubble size and can dampen the movement of hollow fibres in submerged bundles [17]. Wicaksana et al. [17] reported that average values of the velocity of bubble rise in higher-viscosity suspensions were significantly lower than in the less viscous ones.

The application of air bubbles (air sparging) reduces the rise in transmembrane pressure (TMP) due to its scouring effect on the deposited layer on the membrane surface for all operating conditions. A more significant reduction in highly viscous feed could be due to the formation of large bubbles in a more viscous suspension [17,18,19]. Large bubbles are beneficial because they have larger wake regions, create stronger secondary flows and are more effective in promoting local mixing [15]. Li et al. [20] similarly reported that a larger bubble size could result in a stronger wake, enhanced local mixing and the mass transfer from the membrane surface back to the suspension.

From the literature, it is evident that membrane fouling is the most severe problem in membrane separation processes caused by the attachment, accumulation and adsorption of substances in the suspended and colloidal particles onto the membrane surface and within the membrane pores. Fouling significantly increases hydraulic resistance, requiring frequent membrane cleaning and replacement, resulting in high operating costs [21]. Fouling can be reversible and irreversible. Reversible fouling is readily removable by an appropriate physical washing process (backwashing); irreversible fouling, however, is non-washable by routine hydraulic backwashing and requires chemical cleaning. Based on materials, fouling could result from inorganic l (scaling), colloidal, microbial/biological and organic material deposition. This study investigated colloidal fouling to understand the effect of operating parameters on membrane fouling irrespective of membrane material [21].

All previous studies were conducted with biomass in a membrane bioreactor, and several biological and physicochemical parameters were found to play a role. The effect of viscosity on fouling was not studied independently. In this study, membrane microfiltration experiments were carried out with a flat sheet membrane module submerged in a kaolin suspension with predetermined glycerol concentrations [21]. Experiments with feeds of varying viscosity were carried out to examine the effect of viscosity on fouling. During the experiments, the air was injected at various rates to evaluate their effect on viscosity and airflow.

## 2. Materials and Methods

Laboratory experiments were performed using a flat sheet membrane module (11.5 × 10.5 × 22.5 cm (width × length × height)) made of polyvinylidene fluoride (PVDF) with a nominal pore size of 0.14 µm and an effective filtration area of 0.2 m^2^ (A3 company, Gelsenkirchen, Germany). The membrane consisted of eight verticle flat sheets equally spaced 12 mm apart. A membrane reactor with a capacity of 12 L was filled with 10 L of suspension prepared from kaolin clay powder (Sigma, St. Louis, MO, USA), varying in size between 0.1–4 µm (average size 2.1 µm) and tap water. The concentration of clay was kept at 10 g/L and similar to the range of MLSS concentration in a membrane bioreactor.

**Figure 1:**
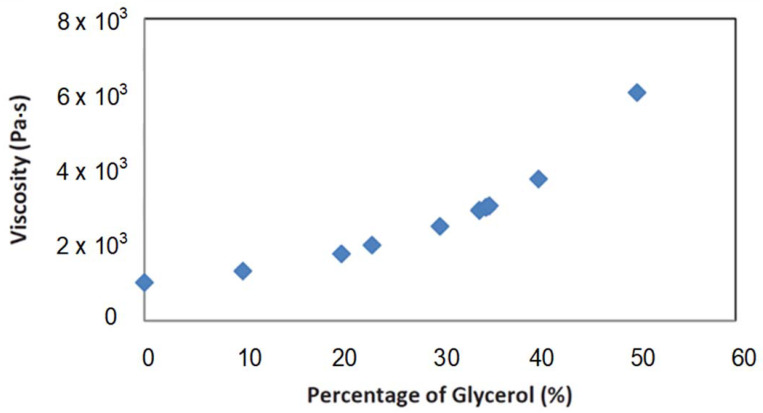
Glycerol fractions for varying concentrations of viscosities.

The feed viscosity of the suspension was varied by adding a predetermined amount of glycerol to the feed solution. Feeds with viscosities of 0.001, 0.002 and 0.003 Pa·s were prepared. Based on data available in the literature [22], a correlation between the viscosity and quantity of glycerol was established to determine the amount of glycerol required for various viscosity of feed. Figure 1 shows the glycerol fraction that needs to be added to the suspension to achieve the different viscosities. This figure was derived from the interpolated values for each glycerol fraction at a temperature of 20 °C [22]. Glycerol refers to the chemical compound 1,2,3-propanetriol and has a chemical formula CH_2_OHCHOHCH_2_OH with a molecular weight of 92.10. It is completely soluble in water and alcohol but is not soluble in ether. At normal temperatures, glycerine remains a viscous suspension up to 100% concentration. Thus, it is available for use over a wide range of viscosities without crystallization. Different concentrations of glycerine were used to obtain solution viscosities of 0.001, 0.002 and 0.003 Pa·s.

**Figure 2:**
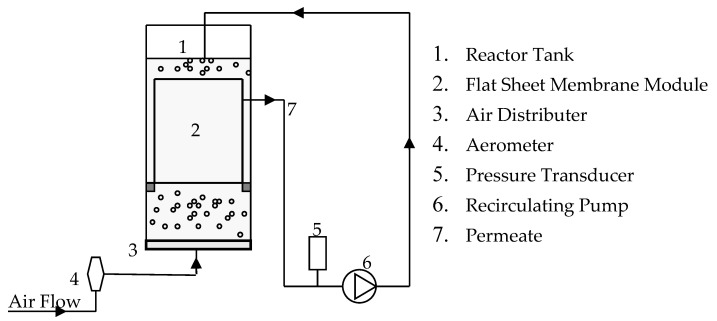
Schematic diagram of the experimental setup.

The schematic diagram of the experimental setup is presented in Figure 2. Laboratory scale microfiltration tests were carried out with a flat sheet membrane. The membrane reactor, which had a capacity of 12 L, was filled with kaolin clay suspension prepared with kaolin clay powder of a mean particle diameter of 2.1 μm. The concentration of the kaolin clay suspension was 10 g per litre (g/L) of tap water. The membrane was submerged in the reactor, and air bubbles at different air flow rates were continuously supplied from the bottom of the reactor, where the air diffuser plate was attached. Firstly, preliminary submerged membrane experiments were conducted with tap water at a filtration rate of 15 L/m^2^/h (viscosity = 0.001, 0.002 and 0.003 Pa·s) without adding kaolin particles to study the effect of viscosity on TMP development. Later experiments were carried out with the addition of kaolin clay (concentration of 10 g/L) at different air flow rates (0.6, 1.2 and 1.8 m^3^/m^2^/h). It should be noted that the airflow rate is expressed in terms of membrane area, and its unit is denoted as m^3^/m^2^/h. The air bubbles were relatively large, with diameters of 2–4 mm.

Before each experiment, the membrane was tested for its hydraulic resistance. The membrane cleaning procedure involved three stages of cleaning. The membrane was first cleaned with tap water. It was then placed in a specially designed holding unit attached to the shaker for an hour at 120 rpm and cleaned with tap water again. Finally, the membrane was submerged in a chemical solution (3% *w*/*w* sodium hypochlorite) for three hours. The process was repeated if the hydraulic resistance had not reduced to that of its virgin state (original).

Particle deposition on the membrane surface was calculated indirectly by measuring the suspended solids concentration. Due to the particle deposition on the membrane, the concentration in the suspension decreased continuously. From the material balance of the particle mass in the whole system, the amount of mass deposited on the membrane was calculated. Samples from the reactor were collected at 1 h intervals, passed through a 0.45 μm filter, and the mass was measured. The TMP was monitored online by a pressure transducer located between the suction pump and the membrane. The use of constant flux mode has proved to be particularly useful in the context of monitoring fouling in complex fluids and is used for membrane bioreactor (MBR) applications [12]. Experiments were carried out at different air flow rates (0.6, 1.2 and 1.8 m^3^/m²/h) and different constant permeates flux rates (10 and 15 L/m^2^/h). A sample from the reactor was collected at 1 h intervals, passed through a 0.45 micrometer filter, and the mass was measured. The particle size distribution was also measured using a particle size zanalyzer (Mastersizer 2000, Malvern WR14 1XZ, UK).

Several experiments were duplicated. The deviation in TMP and particle deposition was less than 2%.

## 3. Results and Discussion

Preliminary continuous experiments of submerged membrane micro-filtration were conducted with different predetermined concentrations of glycerol mixed in tap water, i.e., without the addition of kaolin clay, to study viscosity’s effect on TMP. At 15 L/m^2^/h, the TMP with clean water was in the range of 2 kPa. After adding glycerol (to make viscosities of 0.002 Pa·s and 0.003 Pa·s), the TMP increased to approximately 4 kPa and 6 kPa, respectively. Apart from the TMP rise due to an increase in viscosity, there was no other influence to cause a rise in TMP during the experiment. Darcy’s law [23] expresses the directly proportional relationship between TMP and viscosity, i.e.,

J = TMP/(μR_t_) (1)

where J is the permeate flux (m^3^/m^2^/h), TMP is the transmembrane pressure (N/m^2^), µ the permeate viscosity (Pa·s), and R_t_ is the total membrane resistance (m^−1^).

Other experiments were conducted to study the influence of change in viscosity of kaolin suspension on fouling of a submerged membrane micro-filter. As the concentration of kaolin was kept constant, the only contributing parameter to cause a rise in TMP was the variation of the viscosity of the suspension. Using the experimental data, fouling was directly correlated to the variation of measured TMP. An increase in TMP corresponded to fouling on the membrane surface; hence cake resistance (R_c_) could be determined from Darcy’s law using the experimental measurement of TMP. The TMP developed over the filtration period was used to calculate the total resistance (R_t_) by the following, which is a rearrangement of Equation (1).

R_t_ = TMP/(μ × J) (2)

The total membrane resistance is the sum of R_c_ and clean membrane resistance (R_m_). Resistance due to pore blocking may be neglected because the diameter of kaolin clay particles was greater than the membrane pore size. R_m_ was constant for all tests (R_m_ = 3.8 × 10^11^ m^−1^); therefore, increases in R_c_ correspond to TMP development during the filtration of feed of different viscosity. According to Darcy’s Equation (2), an increment in TMP due to a change in viscosity at a constant filtration flow causes the same increment in total resistance.

Particle deposition on the filter was measured at regular intervals during the experiment. The relationship between R_c_ and particle deposition was analyzed, and specific cake resistance was evaluated for different operating conditions. The cake resistance can be used to calculate the cake mass (w_c_) following Equation (3) [21]. In this study, experiments were carried out in the presence of air scour.

R_c_ = w_c_ × α_av_ (3)

where α_av_ is an average specific membrane resistance.

### 3.1. Effect of Viscosity on R_c_

**Figure 3:**
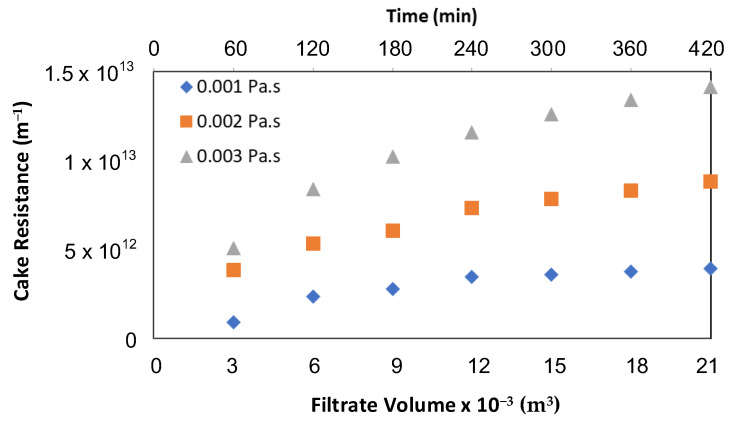
Effect of viscosity on R_c_ at different concentrations of viscosity with an airflow rate of 1.2 m^3^/m^2^/h.

Figure 3 shows the behavior of R_c_ for viscous suspensions at 15 L/m^2^/h with an airflow rate of 1.2 m^3^/m^2^/h. The pattern of development of R_c_ was similar to that observed with filtered water for all operating conditions. The figure shows a sharp rise of R_c_ at the beginning, followed by a slow increase and finally a plateau (steady-state). The trend of the R_c_ curves was similar for all three viscosities for the air flow rates that were used. The value of R_c_ at the steady-state depends on the airflow rate. The application of higher airflow resulted in lower R_c_. Pradhan et al. [24,25,26] also observed similar results during the submerged membrane microfiltration process. Changes in viscosity of the suspension played a significant role in the cake layer formation, resulting in a higher R_c_ for high viscous feed.

The change in R_c_ for the different concentrations of viscosity was assessed based on the volume of filtrate water at the end of the run period (7 h). At a permeate flux of 15 L/m^2^/h, where viscosity was doubled (from 0.001 Pa·s to 0.002 Pa·s), R_c_ increased by 2.31 and 2.22 fold at airflow rates of 0.6 and 1.8 m^3^/m^2^/h, respectively, compared to the result obtained with the base viscosity of 0.001 Pa·s (base case). Similarly, in the case of tripling the viscosity (at 0.003 Pa·s), R_c_ increased by 4.0, 3.4 and 3.22 fold at airflow rates of 0.6, 1.2 and 1.8 m^3^/m^2^/h, respectively, compared to the R_c_ of the base case. These results show that, based on the collection of the same volume of filtrate water, the filtration system had a higher R_c_ with a more viscous kaolin suspension. However, increased air flow helped to reduce this R_c_ even in a viscous medium. It is noted that at a particular permeate flux, the effect of viscosity was larger than predicted by Darcy’s law. This was because an increase in viscosity increases the fouling rate. The influence of viscosity was found to be more important at a low air flow rate.

Before adding glycerol, tripling the airflow (to 1.8 m^3^/m^2^/h) at a permeate flux of 15 L/m^2^/h reduced R_c_ by 60%, whereas doubling the airflow (to 1.2 m^3^/m^2^/h) reduced it by 25% compared to a base airflow of 0.6 m^3^/m^2^/h (base case). When feed viscosity was 0.002 Pa·s, R_c_ reductions were 20% and 62% with doubled and tripled airflow, respectively, compared to the base case. In the case of 0.003 Pa·s, doubling the airflow reduced R_c_ by 29%, whereas a 3-fold increment in air flow caused a 65% reduction compared to the base case. These results show that higher air flows were more effective for highly viscous solutions. The data highlight that increased airflow reduces R_c_ even at high viscous feed suspension. A more significant reduction in highly viscous feed could be due to the formation of larger bubbles in a more viscous suspension. Wicaksana et al. [17] found that the bubble sizes in more viscous suspensions were larger than those in less viscous solution (water). Similar results were observed by Schafer et al. [19]. Large bubbles have larger wake regions and create stronger secondary flows. They are more effective not only in promoting local mixing but also in scouring the cake layer deposited on the membrane surface. These air bubbles help to reduce TMP and particle deposition, and consequently, R_c_.

Viscosity (μ) is explicitly incorporated in Darcy’s equation (Equation (1)) and increases with glycerol solute concentration. TMP depends on viscosity and total membrane resistance for a constant permeate flux. The experimental results obtained by varying viscosity and airflow rates indicated that R_c_ was directly proportional to the viscosity of the suspension and inversely related to the system airflow rates. Doubling the viscosity produced more than double R_c_ for all the airflow rates. In the case of tripling viscosity, low airflow (0.6 m^3^/m^2^/h) resulted in a 4-fold increase in R_c_, whereas high air flow rates (1.8 m^3^/m^2^/h) limited this to 3.22 fold. The effect of viscosity on R_c_ at different viscosity with a lower and higher airflow rate of 0.6 and 1.8 m^3^/m^2^/h is given in the Supplementary Material (Figures S1 and S2). They apply to different permeate fluxes of 10 and 15 L/m^2^/h.

### 3.2. Effect of Viscosity on Particle Deposition (Fouling)

**Figure 4:**
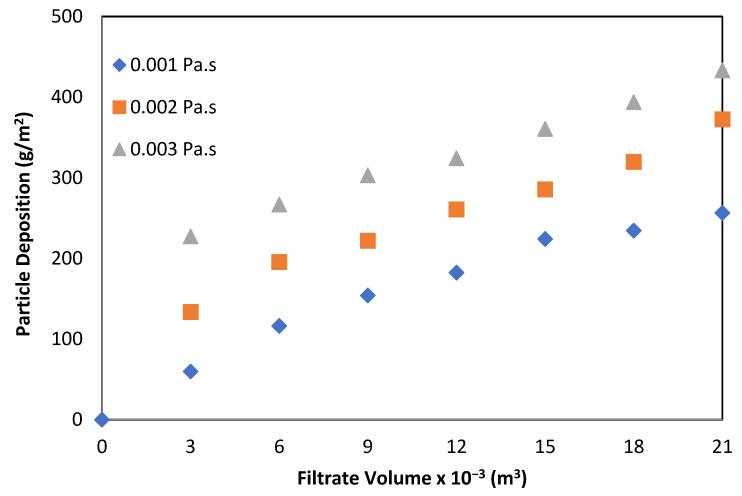
Effect of viscosity on particle deposition on membrane surface at different concentrations of viscosity with air flow rate of 1.2 m^3^/m^2^/h.

The particle deposition on the membrane surface was assessed at a permeate flux rate of 15 L/m^2^/h and by varying both the airflow rate (0.6, 1.2 and 1.8 m^3^/m^2^/h) and viscosity. Figure 4 shows the effect of viscosity on particle deposition at an airflow rate of 1.2 m^3^/m^2^/h and permeate flux of 15 L/m^2^/h. At this flux, an increased viscosity resulted in a higher deposition on the membrane surface for all air flow rates, although an apparent difference was observed at the highest air flow rate of 1.8 m^3^/m^2^/h. For all operating conditions, an increased air flow helped to reduce the particle deposition during submerged microfiltration.

It is clear from Figure 3 that the particle deposition was rapid in the first hour of membrane operation, especially in more viscous solutions (i.e., 0.003 Pa·s). Deposition rose very sharply during the first hour of the experiment for all operating conditions, where almost half (48%) of the deposition took place. This indicates how critical the initial stage of membrane operation is to membrane fouling. Special attention to control fouling during the initial period of filtration could result in better membrane operation.

### 3.3. Relationship between R_c_ and Particle Deposition

In general, the increment in TMP corresponds to an increase in R_c,_ which is directly related to the increase in particle or cake deposition on the membrane surface. Therefore, many researchers have zanalyzed membrane fouling in terms of TMP. In this study, both these parameters (TMP and cake deposition) were measured experimentally, and R_c_ was calculated using Darcy’s law to establish a relationship between R_c_ and cake deposition. The relationship was regressed and compared to Equation (3) to determine the average specific cake resistance (α_av_) at different operating conditions.

**Figure 5:**
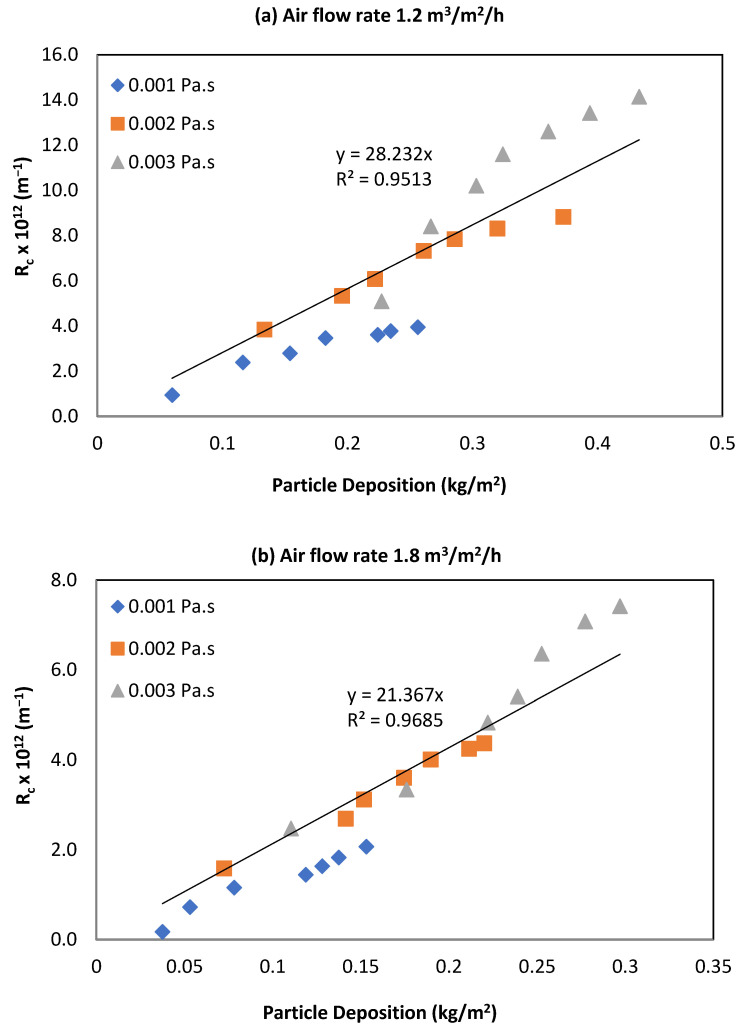
Relationship between R_c_ and particle deposition at 15 L/m^2^/h and two airflow rates.

Figure 5a,b show the relationship between the R_c_ and cake deposition at different viscosities and airflow rates for a permeate flux of 15 L/m^2^/h. The relationship followed an almost linear pattern for kaolin clay suspension for all viscosities that were tested. All operating conditions experienced higher cake deposition with high R_c_ and viscosity. An increased airflow led to a reduction in both R_c_ and cake deposition. The regressed equations show a significant reduction in α_av_ with increased air flow rate.

The figures showing the relationship between the R_c_ and cake deposition at different viscosities and airflow rates for a permeate flux of 10 and 15 L/m^2^/h are shown in the Supplementary Material (Figures S3 and S4).

From Table 1, it is clear that the effect of high air flow is more effective at high viscosity. At 10 L/m^2^/h, in the absence of glycerol (0.001 Pa·s), tripling the air flow rate (from 0.6 to 1.8 m^3^/m^2^/h) caused a 51% reduction in cake resistance, while at solution viscosities of 0.002 Pa·s and 0.003 Pa·s, the reductions were 59% and 66%, respectively. At a higher flux of 15 L/m^2^/h, cake resistance reduced by 60%, 62%, and 65% for corresponding viscosities of 0.001, 0.002, and 0.003 Pa·s under the same operating conditions mentioned above (tripling airflow). These results show that cake resistance reduction is more significant at high airflow and high viscous feed than at low airflow and less viscous feed.

**Figure 6:**
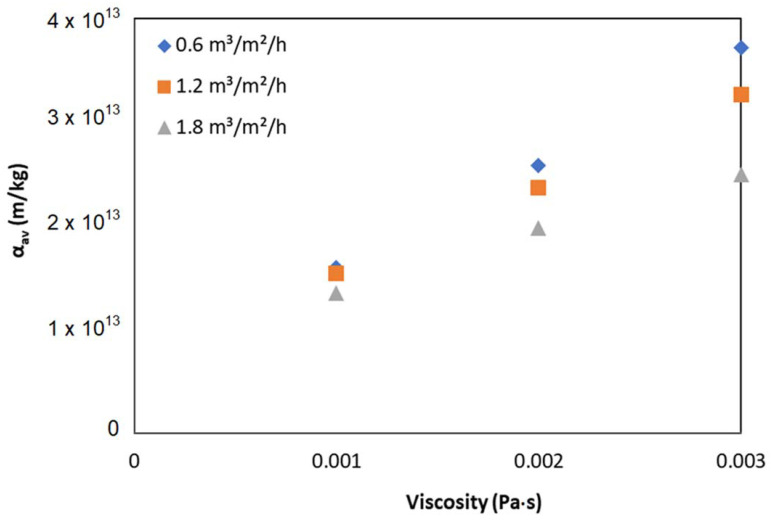
Effect of viscosity on specific filtration resistance.

Figure 6 shows the effect of viscosity on α_av_ at a permeate flux of 15 L/m^2^/h. Compared to the impact of airflow, an increase in viscosity significantly increased the α_av_.

## 4. Conclusions

The results indicate that the feed viscosity is among the more important influencing parameters that affect membrane fouling. It has not been studied previously under controlled conditions. In a membrane bioreactor, similar to conventional activated sludge processes, biomass viscosity is closely related to the concentration of mixed liquor suspended solids (MLSS) and has been referred as a foulant parameter. In wastewater applications, a high concentration of MLSS in the reactor results in high viscosity.

The influence of changing viscosity was investigated by adding predetermined quantities of glycerol to a kaolin clay suspension during submerged membrane microfilter operating in the presence of air flow bubbles. It was demonstrated that the viscosity of the suspension played a significant role in membrane fouling. An increase in viscosity caused a significant rise in TMP, and particle deposition, leading to the formation of higher cake resistance (R_c_). The application of air scour helped to reduce the TMP and particle deposition on the membrane surface. The airflow/scour is the most important practical parameter that controls the membrane fouling in a viscous feed solution. At a permeate flux of 10 L/m^2^/h, tripling the aeration rate caused a 51% reduction in cake resistance, while at feed viscosities of 0.002 Pa·s and 0.003 Pa·s, the reductions were 59% and 66%, respectively.

The relationship between R_c_ and particle deposition was approximately a linear correlation for various suspension viscosity and applied airflow rates. The specific filtration resistance (α_av_) reduced with the application of higher airflow but increased with higher feed viscosity.

Conversely, an increase in feed viscosity aggravated membrane fouling. Due to the increased viscosity, the increment in cake resistance was far greater than the reduction in resistance caused by the increased airflow. Moreover, the cake resistance demonstrated a strong dependency on the permeate flux even in a viscous suspension.

## Figures and Tables

**Table 1:** Effect of air on cake resistance reduction (%) with varying feed viscosity.

Flux (L/m^2^/h)	Feed Viscosity: 0.001 Pa·s			Feed Viscosity: 0.002 Pa·s			Feed Viscosity: 0.003 Pa·s		
	Airflow Increment (m^3^/m^2^/h)			Airflow Increment (m^3^/m^2^/h)			Airflow Increment (m^3^/m^2^/h)		
	0.6–1.2	0.6–1.8	1.2–1.8	0.6–1.2	0.6–1.8	1.2–1.8	0.6–1.2	0.6–1.8	1.2–1.8
10	33	51	31	28	59	45	42	66	45
15	25	60	46	20	62	51	29	65	50

## Data Availability

Not applicable.

## Supplementary Materials

The following supporting information can be downloaded at: https://www.mdpi.com/article/10.3390/membranes12080780/s1. Figure S1: Effect of viscosity on cake resistance under various air flow rates at 10 L/m^2^/h (Kaolin clay concentration = 10 g/L); Figure S2: Effect of viscosity on cake resistance under various air flow rates at 15 L/m^2^/h, (Kaolin clay concentration = 10 g/L); Figure S3: Effect of viscosity on particle deposition under various air flow rates at 10 L/m^2^/h; Figure S4: Effect of viscosity on particle deposition at a permeate flux of 15 L/m^2^/h.

## Abbreviations

EPS	Extracellular polymeric substance
J	Permeate flux
MLSS	Mixed liquor suspended solids
Pa·s	Pascal-second
R_t_	Total membrane resistance
R_c_	Cake resistance
R_m_	Membrane resistance
rpm	Rotation per minutes
TMP	Transmembrane pressure
µm	Micro meter
µ	Viscosity
wc	Cake mass
αav	An average specific membrane resistance
