# Supplementary material for: Effects of Viscosity on Submerged Membrane Microfiltration Systems
Source: Membranes (Basel). 2022 Aug 14;12(8):780. doi: 10.3390/membranes12080780 (PMC9413319; doi:10.3390/membranes12080780)
Supplement: Supplementary file 1 [file membranes-12-00780-s001.zip › membranes-1836780-supplementary.pdf]

Supplementary Material

Effects of Viscosity on Submerged Membrane Microfiltration Systems

Muna Pradhan[1, 2], Md Abu Hasan Johir[1], Jaya Kandasamy[1], Harsha Ratnaweera[3], Saravanamuthu Vigneswaran [1, 3*]

1 Faculty of Engineering, University of Technology Sydney (UTS), P.O. Box 123, Broadway, 1 Faculty of Engineering, University of Technology Sydney (UTS), P.O. Box 123, Broadway, NSW 2127, Australia

2 BMB Engineers, P.O. Box 7842, Baulkham Hills, NSW 2153, Australia

3 Faculty of Sciences & Technology (RealTek), Norwegian University of Life Sciences, P.O. Box 5003, NO-1432 Ås, Norway

* Correspondence: saravanamuth.vigneswaran@uts.edu.au

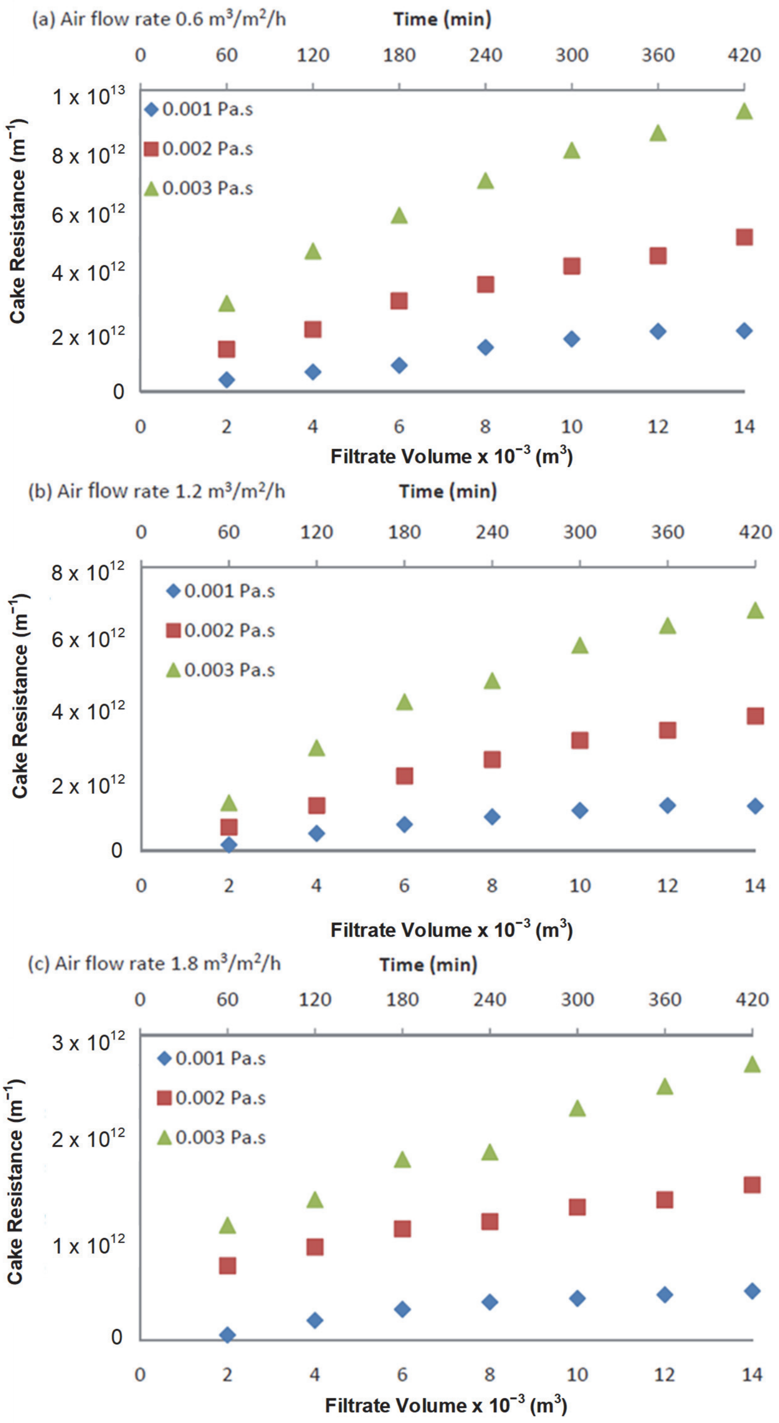

Figure S1: Effect of viscosity on cake resistance under various air flow rates at 10 L/m²/h (Kaolin clay concentration = 10 g/L)

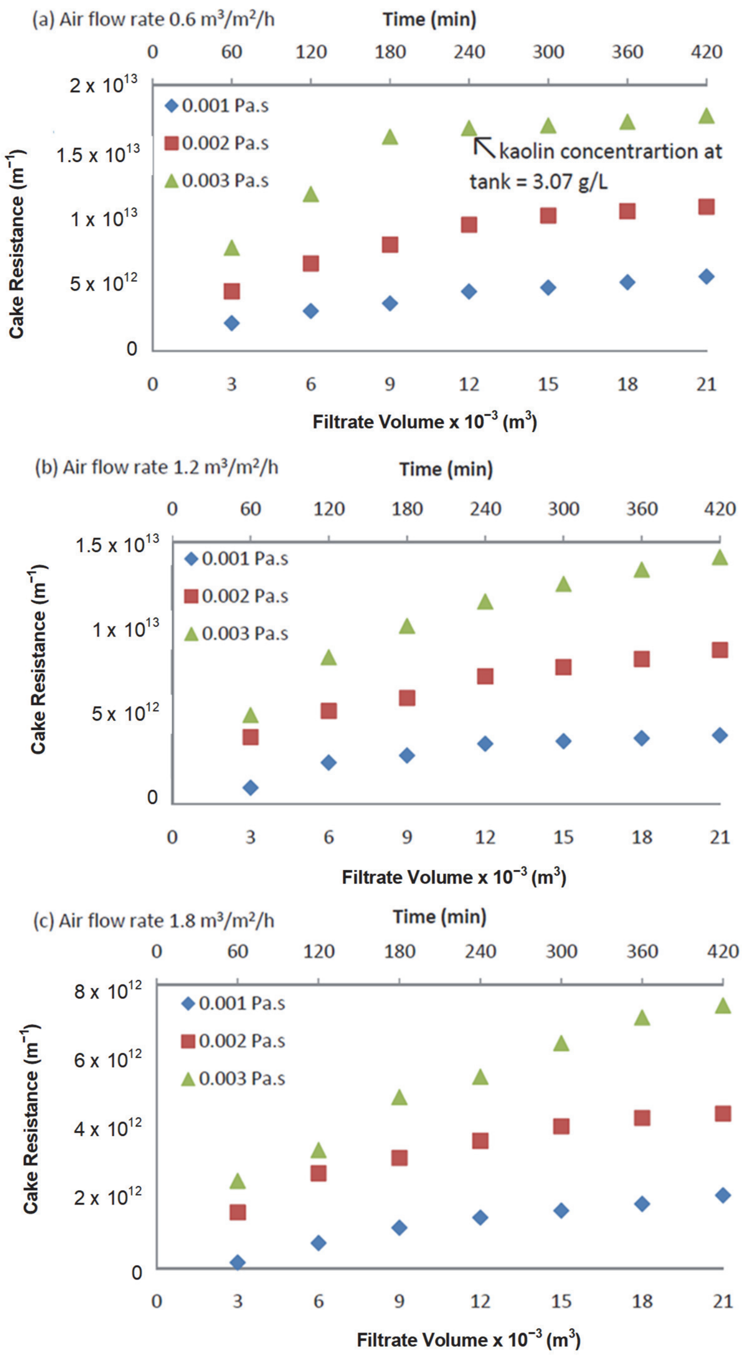

Figure S2: Effect of viscosity on cake resistance under various air flow rates at 15 L/m^2/h, (Kaolin clay concentration = 10 g/L)

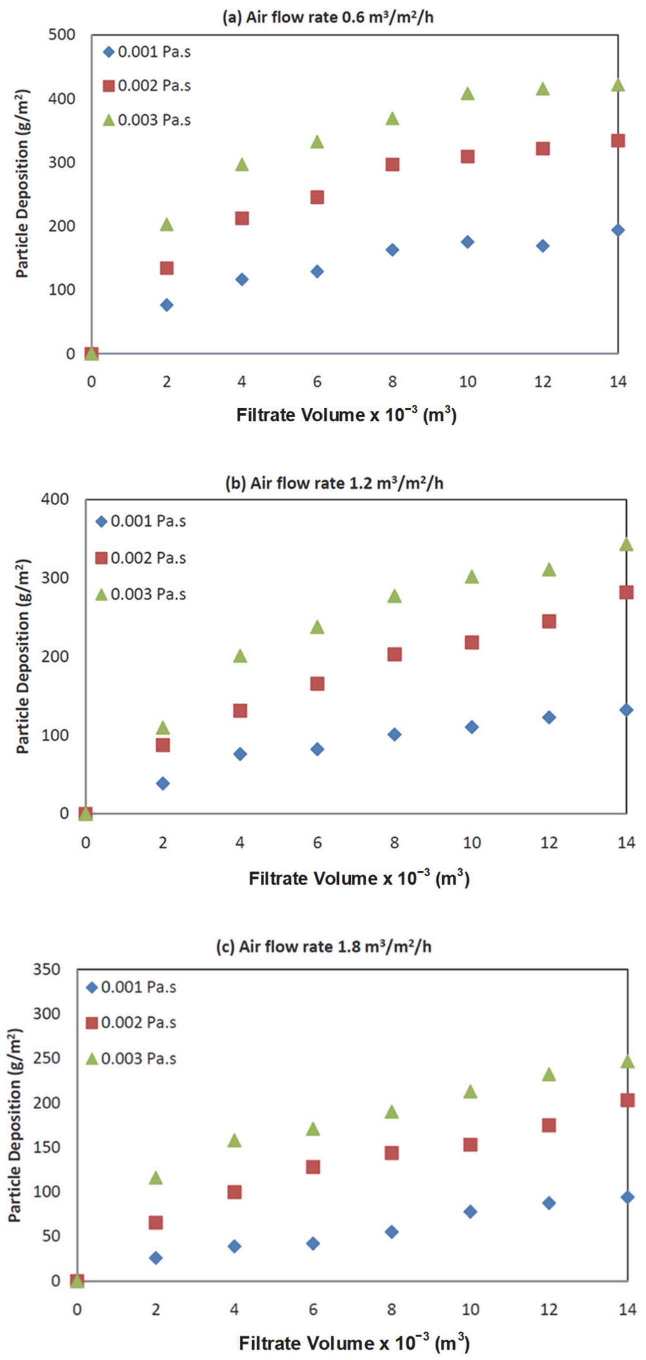

Figure S3: Effect of viscosity on particle deposition under various air flow rates at 10 L/m^2/h

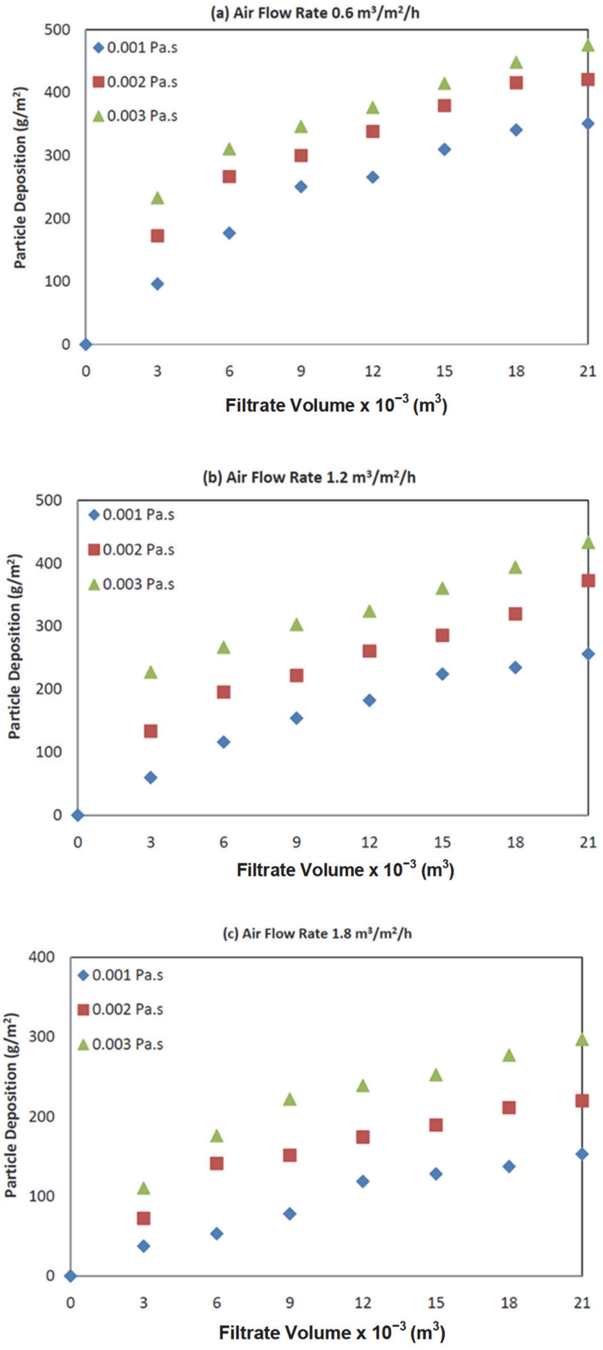

Figure S4: Effect of viscosity on particle deposition at a permeate flux of 15 L/m^2/h
